# Absence of neutralizing antibodies against the Omicron SARS-CoV-2 variant in convalescent sera from individuals infected with the ancestral SARS-CoV-2 virus or its Gamma variant

**DOI:** 10.1016/j.clinsp.2022.100068

**Published:** 2022-06-16

**Authors:** Lucy Santos Villas-Boas, Anderson Vicente de Paula, Almir Ribeiro da Silva, Heuder Gustavo Oliveira Paiao, Tania Regina Tozetto-Mendoza, Erika Regina Manuli, Fábio Eudes Leal, Andrea de Barros Coscelli Ferraz, Ester Cerdeira Sabino, Ana Luiza Bierrenbach, Steven Sol Witkin, Maria Cassia Mendes-Correa

**Affiliations:** a Instituto de Medicina Tropical de Sao Paulo, Faculdade de Medicina da Universidade de Sao Paulo, São Paulo, SP, Brazil; b Departamento de Moléstias Infecciosas e Parasitarias da Faculdade de Medicina da Universidade de São Paulo, São Paulo, SP, Brazil; c Faculdade de Medicina da Universidade Municipal de Sao Caetano do Sul, São Caetano, SP, Brazil; d Instituto de Ensino e Pesquisa do Hospital Sírio-Libanês, São Paulo, SP, Brazil; e Weill Cornell Medicine, USA

**Keywords:** SARS-CoV-2, Omicron variant, Gamma variant, Neutralizing antibodies, Protective antibodies

## Abstract

**Objectives:**

The aim of the present study was to evaluate if neutralizing antibody responses induced by infection with the SARS-CoV-2 strain that was dominant at the beginning of the pandemic or by the Gamma variant was effective against the Omicron variant.

**Methods:**

Convalescent sera from 109 individuals, never exposed to a SARS-CoV-2 vaccine, who had mild or moderate symptoms not requiring hospitalization following either a documented SARS-CoV-2 ancestral strain infection or a Gamma variant infection, were assayed for *in vitro* neutralizing antibody activity against their original strains and the Omicron variant.

**Results:**

Following an infection with the ancestral strain, 56 (93.3%), 45 (77.6%) and 1 (1.7%) serum sample were positive for neutralizing antibodies against the ancestral, Gamma variant, and Omicron variant, respectively. After infection with the Gamma variant, 43 (87.8%) and 2 (4.1%) sera were positive for neutralizing antibodies against the Gamma and Omicron variants, respectively.

**Conclusions:**

Neutralizing antibodies generated following mild or moderate infection with the SARS-CoV-2 ancestral strain or the Gamma variant are not protective against the Omicron variant.

## Highlights

• Individuals previously infected with SARS-CoV-2 ancestral strain do not develop neutralizing antibodies against the Omicron variant.
• Omicron variant escapes immune response after SARS CoV-2 previous infection with the SARS CoV-2 Gamma variant.
• Individuals previously infected with SARS-CoV-2 ancestral strain or with SARS-CoV-2 Gamma variant will likely have little protection if subsequently exposed to the Omicron variant.

## Introduction

Since the beginning of the Coronavirus Disease 2019 (COVID-19) pandemic, one of the major concerns has been the duration and specificity of immune protection following initial infection. The long-term clinical and immunological consequences of anti-viral antibody production against the infecting strain remain unclear and correlations between antibody levels and protection against re-infection by new SARS-CoV-2 variants remain under-reported [1,2].

Brazil has been heavily affected by the COVID-19 epidemic and has experienced multiple waves of infection since its initial identification in February 2020, almost two months after the announcement of its outbreak in China. Beginning in November 2020, a novel SARS-CoV-2 variant, Gamma variant (P.1), was identified in Manaus, Brazil [3]. Until July 2021 it was the most frequent variant sequenced in this country [4]. Beginning in December 2021 and continuing until the present time the Omicron variant has spread throughout Brazil and now accounts for 85% of all sequenced cases [4].

Neutralizing antibodies react with surface components of SARS-CoV-2, specifically the spike protein, and prevent the virus from interacting with specific receptors on target cells and thereby initiating a productive infection [1]. They also contribute to protection from reinfection [5,6]. Genome sequencing has demonstrated that the Gamma and Omicron variants are characterized by multiple mutations in the gene coding for the spike protein. These mutations result in antigenic changes that could impair the efficacy of neutralizing antibodies that were generated against a previous SARS-CoV-2 strain [7]. Measuring and comparing the neutralization capacity of antibodies in sera from convalescent individuals previously infected with SARS-CoV-2 strains circulating at the beginning of the pandemic with genetic variant strains present at late pandemic stages will provide much-needed information regarding the occurrence of cross-immunity between different viral strains.

The aim of the present study was to evaluate if neutralizing antibody responses were induced by infection with the SARS-CoV-2 virus that was dominant at the beginning of the pandemic or induced by the Gamma variant, which was first described in Brazil and remained as a dominant SARS-CoV-2 variant in the country for almost a year [4], remained effective when tested against the Omicron variant.

## Methods

### Setting and patients

Included patients were participants in The Corona São Caetano Program, a primary care initiative offering COVID-19 care to all residents of São Caetano do Sul, Brazil [8]. Sixty participants who were positive for the SARS-CoV-2 ancestral strain (Group 1) and 49 participants who were positive for the Gamma variant (Group 2) were enrolled in this study. All had a confirmed SARS-CoV-2 infection by RT-PCR analysis of nasopharyngeal swabs (QIAamp viral RNA kit and RealStar® SARS-CoV-2 RT-PCR Kit 1.0, developed by Altona Diagnostics).

Complete viral genomes in all samples were generated using the MinION sequencing platform (Oxford Nanopore Technologies, ONT, UK) as previously described [9]. All sequenced samples were classified as belonging to either the ancestral lineage or to the Gamma variant [10].

All samples from participants in Group 1 were collected between May 4 and May 16, 2020, (months before the first recorded infections associated with the Gamma lineage in Brazil and months before any SARS-CoV-2 vaccines were available). Samples from participants in Group 2 were collected between April 12th and June 25th, 2021, and none of them received a SARS-CoV-2 vaccine before inclusion in the study. After obtaining written informed consent, peripheral blood for serological analysis was collected from each participant.

### Virus isolation and titration for virus neutralization test

#### Virus isolation

An ancestral variant (EPI_ISL_1557222) which was classified as belonging to the B.1.1.28 lineage, was cultured from a nasopharyngeal sample taken from an infected patient from Sao Caetano do Sul, City, Brazil in April 2020. The Gamma SARS-CoV-2 variant (EPI_ISL_1060902) was obtained from a nasopharyngeal specimen of a patient from Manaus City, Brazil, in December 2020 that was previously classified as belonging to the Gamma lineage by virus genome sequencing [3]. The Omicron variant (EPI_ISL_6901961) was generously provided to us by Professor Edison Durigon from Instituto de Ciencias Biológicas ‒ USP.

To isolate SARS-CoV-2 the authors used Vero cells (ATCC® CCL-81™). Cells were seeded in a cell culture flask (polystyrene sterile, non-pyrogenic flask, 12.5 cm^2^, 25 mL, Biofil®, China) at a concentration of 2 × 10^5^ cells/mL in 3.0 mL Dulbecco Minimal Essential Medium (DMEM) supplemented with 5% heat-inactivated Fetal Bovine Serum (FBS) (Vitrocell Embriolife, Campinas, Brazil) and incubated overnight at 37°C. The next day, the supernatant was discarded, and 0.5 mL of a homogenized viral specimen was added to the culture flask. After 1h of incubation (adsorption), the authors supplemented the volume with 3.0 mL DMEM containing 2.5% FBS and 1% penicillin-streptomycin and incubated the cultures in a humidified 37°C incubator in an atmosphere of 5% CO_2_. The authors observed the Presence of Cytopathic Effects (CPE) daily for 3‒5 days. The supernatant was collected, and virus replication was confirmed through CPE and by RT-PCR (QIAamp viral RNA kit and RealStar® SARS-CoV-2 RT-PCR Kit 1.0, by Altona Diagnostics- target E and S genes). The whole-genome sequence of the culture isolates was determined using nanopore sequencing (Oxford Nanopore Technologies). Purified libraries were sequenced with the Oxford Nanopore MinION device using R9.4.1 flow cells.

#### Virus titration

Twenty-four hours before viral addition, the authors prepared 96 well sterile polystyrene microtiter plates (Jet Bio-Fil- China) containing 5 × 10^4^ cells/mL Vero CCL-81 cells in DMEM. A virus preparation was 10-fold serially diluted in medium (10^−1^ to 10^−12^), the original culture medium was then removed and replaced with serial dilutions of the virus in sextuplicate wells and incubated at 37°C. Observations were performed daily using an inverted light microscope (Nikon 45178, Japan) to verify the presence of CPE over a 72h period. The monolayers were then fixed and stained with Naphthol Blue Black (Sigma-Aldrich Co., Deisenhofen, Germany) dissolved in sodium acetate-acid acetic. The viral titer was expressed in TCID_50_/mL and calculated using the Spearman & Kärber algorithm, as described by Hierholzer & Killington [11].

#### Virus Neutralization Test (VNT)

The Cytopathic Effect (CPE)- based Virus Neutralization Test (VNT) was adapted from Nurtop et al. [12] and was previously described by Wendel et al. [13] and Mendrone-Junior et al. [14]. VNT was performed with SARS-CoV-2 (EPI_ISL_1557222, EPI_ISL_1060902, and EPI_ISL_6901961) in 96-well microtiter plates containing 5 × 10^4^ Vero cells/mL. Vero cells were seeded in a 96-well microtiter plate and allowed to grow for 24 h prior to infection. Sera to be tested were heat-inactivated for 30 min at 56°C. Then, 110 μL of twofold serially diluted sera from 1:20 to 1:2560, were added to mixed vol/vol with 10^3^ TCID_50_/mL of SARS-CoV-2 and incubated at 37°C for 1h for virus neutralization. The sera-virus mixture was transferred onto the confluent Vero cell monolayer and incubated for 72 h. Cultures at 37°C and 5% CO_2_ were observed daily for a CPE. After 72 h, the plates were analyzed by light microscopy (Nikkon, Japan), distinguishing the presence/absence of CPE-VNT. To verify the initial observations after 72 h the monolayers were fixed and stained with Naphthol Blue Black (Sigma-Aldrich Co., Deisenhofen, Germany) dissolved in sodium acetate-acid acetic for 30 min. Dilutions of serum associated with CPE were considered a negative result. The absence of CPE or complete neutralization of SARS-CoV2 inoculum was considered a positive result. Consequently, the VNT was the highest dilution of serum that neutralized viral growth (absence of a CPE).

For each reaction, the authors used as a positive control diluted virus in DMEM with 2.5% FBS and as a negative control only DMEM with 2.5% FBS. In addition, positive control was a serum specimen taken from a patient with a SARS-CoV-2 infection in São Paulo, and a negative control sample from a patient without neutralizing antibodies, with known VNT results. The antibody titer was calculated as the highest dilution where CPE was completely inhibited. Titers ≥1:20 were reported as positive. Virus isolation and VNT were performed in a Biosafety Level 3 laboratory.

### Statistical analysis

The number of sera with neutralizing activity as well as the neutralization titer was described as summary measures for each variant and group. To verify associations between the number of sera with neutralizing activity and the neutralizing titer for each variant following the two infections the authors used the Chi-Square test or Fisher's exact test and Generalized Estimation Equations (GEE) with binomial distribution and logit link function or GEE with gamma distribution and identity link function. Exchangeable matrix correlations between variants and their analyses were followed by Bonferroni's multiple comparisons as necessary [15]. The analyzes were performed using the IBM-SPSS for Windows version 22.0 software and tabulated using the Microsoft-Excel 2010 software.

## Results

The majority of subjects were female (66.9%) and their ages ranged from 18 to 86 with a median of 45 years. The mean time from onset of symptoms to blood collection for participants in both groups was 20.6±2.7 days. All individuals had mild or moderate symptoms that did not require hospitalization.

Table 1 shows that 93.3% of sera from individuals infected with the ancestral strain were positive for *in vitro* neutralizing activity against the ancestral strain, 77.6% were positive against the Gamma variant while only 1.7% possessed neutralizing activity against the Omicron variant (p < 0.001 vs. ancestral and Gamma strains). In individuals who were infected with the Gamma variant, 87.8% had neutralizing activity against Gamma while only 4.1% were able to neutralize the Omicron variant (p < 0.001).

**Table 1:** Number of sera with neutralizing activity against the SARS-CoV-2 Omicron variant in individuals previously infection with the ancestral variant or with the Gamma variant.

Infection	Variant	N° positive/N° tested (%)
Ancestral	Ancestral	56/60 (93.3%)
	Gamma	45/58 (77.6%)
	Omicron	1/60 (1.7%)^a^
Gamma	Gamma	43/49 (87.8%)
	Omicron	2/49 (4.1%)^a^

Sera from individuals infected with the ancestral or Gamma variant of SARS-Co-V2 were tested for *in vitro* neutralizing activity against the ancestral strain and the Gamma and Omicron variants.

a p < 0.001 vs. other variants.

The neutralizing antibody titers against the SARS-CoV-2 strains in sera from individuals infected with the ancestral or Gamma strains are shown in Table 2. Among those infected with the ancestral strain the median neutralizing titer was 1:240 against the ancestral strain, 1:120 against the Gamma variant, and 1:20 against Omicron (p < 0.001). Similarly, in those infected with the gamma variant, the median neutralizing antibody titer was 1:160 against gamma as opposed to 1:20 against Omicron (p < 0.001).

**Table 2:** Titer of neutralizing activity against the SARS-CoV-2 Omicron variant in sera from individuals following infection with the ancestral virus or the Gamma variant.

Infection	Variant	Median titer (interquartile range)
Ancestral	Ancestral	1:240 (1:80, 1:640)
	Gamma	1:160 (1:80, 1:320)
	Omicron	1:20 (1:20, 1:20)^a^
Gamma	Gamma	1:160 (1:40, 1:320)
	Omicron	1:20 (1:20, 1:20)^a^

The neutralizing titer of antibody in sera from individuals infected with the ancestral or Gamma variant of SARS-Co-V2 were determined against the ancestral strain and the Gamma and Omicron variants.

a p < 0.001 vs. other variants.

## Discussion

In convalescent sera from individuals infected with the ancestral or Gamma SARS-CoV-2 strains who exhibited symptoms that did not require hospitalization, *in vitro* neutralizing antibody activity against the Omicron variant was absent in all but one or two individuals and the titers were of borderline detectability. These findings confirm and reinforce prior investigations that Omicron is not neutralized by sera from an individual previously infected with the ancestral, Alpha, Beta, Gamma, and Delta strains of the virus 16, 17, 18, 19. The authors also report the novel finding that sera from 77.6% of individuals infected with the ancestral strain were able to neutralize the Gamma variant, although at reduced titer compared to activity against the ancestral strain.

It is most likely that the observed immune evasion exhibited by Omicron in the present study is due to reported variations in the conformation of the spike protein that reduces the efficacy of antibody binding [7,20]. The receptor-binding domain of the SARS-CoV-2 spike protein is the main target of neutralizing antibodies [21]. Mutations, especially when arising in the S-gene coding for the Spike (S) protein, may affect both viral entry into target cells mediated by the binding of S to its ACEII receptor and antibody efficacy [22].

The huge number of individuals infected during this pandemic and the high viral load produced during each infection offer many opportunities for SARS-CoV-2 mutations to arise and undergo selection. Beginning in November 2020, the Gamma variant was identified in Manaus, Brazil [3]. Genome sequencing demonstrated that this variant was characterized by 17 mutations, including three in the gene coding for the spike protein (K417T, E484K, and N501Y). The Omicron variant, reported by the World Health Organization (WHO) on November 24, 2021, has 32 changes in the amino acid sequence in the spike protein relative to that of the original virus (Wuhan-hu-1), particularly in the receptor-binding domain and the N-terminal domain, the primary targets of neutralizing antibodies [23]. Omicron has shown higher transmissibility leading to many reported breakthroughs and re-infections across the globe, including in Brazil. According to the latest Brazilian Health Ministry epidemiological report, the number of coronavirus-related hospitalizations and deaths rose 149% between December 2021 and January 2022 [24].

It is important to acknowledge the limitations of the present study. First, the cohort included a relatively small number of patients, all of whom recovered from mild or moderate episodes of COVID-19. The level of severity of COVID-19 infection could be associated with different levels of immune memory and subsequent immune protection. It remains to be determined if similar results would be observed in sera from individuals following severe forms of COVID-19 [25,26]. In addition, the present study only assessed neutralizing antibody titers in individuals at a single time point, a mean of 20 days after the initial infection. Even though many studies have suggested that serological neutralizing antibody responses peak within 3–5 weeks after infection, the length of time that the neutralizing antibody titers are maintained remains to be determined [25,26]. Data on humoral immunity to other human coronaviruses have indicated that antibody levels wane over time [2]. Extending this study to later time points would be valuable to gain this additional information. The present study only measured *in vitro* neutralizing antibody activity and did not assess other components of the immune response, such as cell-mediated immunity, which would further contribute to viral neutralization *in vivo*. Large prospective epidemiologic studies will be required to establish the role of neutralizing antibodies in preventing infection, or minimizing their consequences, by SARS-CoV-2 variants in previously exposed individuals.

In conclusion, the present data suggest that the SARS-CoV-2 Omicron variant will not be inhibited by neutralizing antibody responses elicited by prior infection with the ancestral and Gamma variant strains. Thus, previously infected individuals will likely have little protection if subsequently exposed to the Omicron variant.

## Ethics

The study was approved by the local ethics committee, Comissão Nacional de Ética em Pesquisa (CONEP), protocol n° CAAE: 30419320.7.0000.0068, dated April 18, 2020) and all subjects provided informed written consent

## Financial support

This study was supported by a research grant from FAPESP (2020/05623-0) and Merck Sharp & Dohme Farmacêutica LTDA-Investigator Studies Program (MISP#60384). The sponsors did not have any involvement in the study design, data analysis, or its interpretation.

## CRediT authorship contribution statement

**Lucy Santos Villas-Boas:** Conceptualization, Writing – review & editing, Supervision, Formal analysis. **Anderson Vicente de Paula:** Conceptualization, Writing – review & editing, Supervision, Data curation. **Almir Ribeiro da Silva:** Data curation. **Heuder Gustavo Oliveira Paiao:** Data curation. **Tania Regina Tozetto-Mendoza:** Data curation. **Erika Regina Manuli:** Data curation. **Fábio Eudes Leal:** Conceptualization. **Andrea de Barros Coscelli Ferraz:** Data curation. **Ester Cerdeira Sabino:** Conceptualization, Writing – review & editing, Supervision. **Ana Luiza Bierrenbach:** Formal analysis. **Steven Sol Witkin:** Conceptualization, Writing – review & editing, Supervision, Formal analysis. **Maria Cassia Mendes-Correa:** Conceptualization, Writing – review & editing, Supervision, Formal analysis, Funding acquisition.

## Declaration of Competing Interest

The authors declare no conflicts of interest.
